# Retrospective analyses evaluating the mortality risk associated with pimavanserin or other atypical antipsychotics in patients with Parkinson disease psychosis

**DOI:** 10.1016/j.prdoa.2024.100256

**Published:** 2024-05-06

**Authors:** Stuart H. Isaacson, Rajesh Pahwa, Fernando Pagan, Victor Abler, Daniel Truong

**Affiliations:** a Parkinson’s Disease and Movement Disorders of Boca Raton, 951 NW 13th Street, Bldg. 5-E, Boca Raton, FL 33486, USA; b Department of Neurology, University of Kansas Medical Center, 2060 W 39th Ave, Kansas City, KS 66103, USA; c Department of Neurology, Georgetown University Medical Center, 3900 Reservoir Rd NW, Washington, DC 20007, USA; d Acadia Pharmaceuticals Inc, 12830 El Camino Real, San Diego, CA 92130, USA; e The Parkinson and Movement Disorder Institute, 9940 Talbert Ave #100, Fountain Valley, CA 92708, USA; f Department of Psychiatry and Neuroscience, University of California Riverside, 900 University Ave, Riverside, CA 92521, USA

## Abstract

**Introduction:**

Parkinson’s disease (PD) is associated with increased mortality risk (MR), reflecting progression of motor and nonmotor symptoms. PD psychosis (PDP), a common nonmotor symptom, increases with prolonged disease and elevates the MR of PD even further. Pimavanserin is the only FDA–approved treatment for PDP. This review summarizes real-world evidence around the MR of patients with PDP treated with pimavanserin versus off-label atypical antipsychotics.

**Methods:**

A PubMed search was conducted using the following search terms: *pimavanserin* AND *antipsychotic* AND *mortality* AND *Parkinson’s disease* AND *psychosis*. Inclusion criteria specified the entry of retrospective, observational, and open-label studies comparing pimavanserin to atypical antipsychotics or untreated controls.

**Results:**

A total of 10 of the 32 articles met inclusion criteria. Among five comparisons of pimavanserin with atypical antipsychotics, two were large (n = 21,719; n = 21,975), representative, Medicare-database studies, which demonstrated comparable or lower all-cause pimavanserin MR. Among three pimavanserin versus control studies, two reported lower or comparable pimavanserin MR and one, long-term care study reported higher MR for pimavanserin versus non-pimavanserin treated patients with unknown PDP status. Two open-label extensions reported pimavanserin mortality rates of 6.45 and 18.8 deaths per 100 patient-years, which are comparable to, or lower than, mortality rates for PD, PDP, and other atypical antipsychotics. Most studies (70 %; 7 of 10) demonstrated pimavanserin’s MR was lower than or similar to other atypical antipsychotics or untreated controls.

**Conclusions:**

Pimavanserin did not increase the MR in PDP. Pimavanserin’s MR appears to be comparable to or lower than other atypical antipsychotics prescribed for PDP, including quetiapine.

## Highlights

• Mortality rate is increased in Parkinson disease psychosis (PDP)
• Pimavanserin does not appear to increase the mortality risk in PDP.
• Pimavanserin’s mortality risk is lower than or comparable to atypical antipsychotics.

## Graphical abstract

**Figure:**
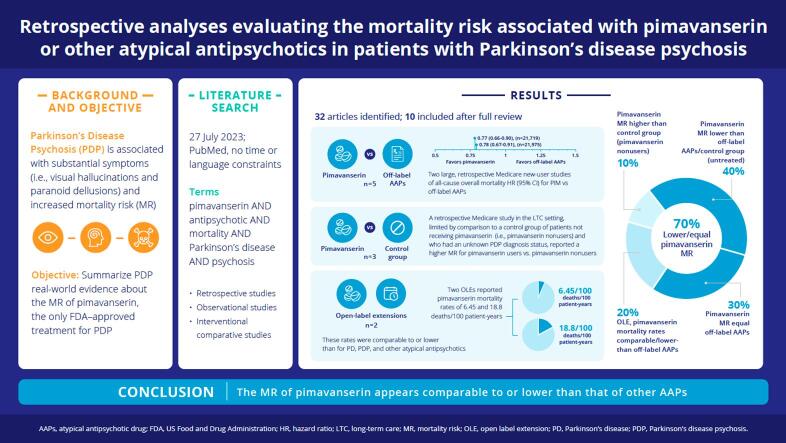

## 1 Introduction

Parkinson disease (PD) is estimated to affect approximately one million adults in the United States [1]. The incidence of PD increases with age, and rates are higher in men, particularly in those aged 60 years and older [2]. Although PD is primarily characterized by motor symptoms, many patients experience a range of nonmotor symptoms, including autonomic dysfunction, sleep disorders, sensory abnormalities, and neuropsychiatric disorders, like PD dementia (PDD) and PD psychosis (PDP) [3], [4]. PDP is common and emerges in more than 50 % of patients with PD progression [5].

PDP development is associated with older age, greater severity and duration of PD, rapid eye movement sleep behavior disorder, orthostatic hypotension, and mutations in the *GBA* gene [4], [6], [7], [8]. PDP is diagnosed when persistent psychosis symptoms (i.e., illusions, false sense of presence, hallucinations and/or delusions) emerge following PD diagnosis, and other causes of psychosis are excluded [9]. Initially, PDP symptoms often include minor phenomena (i.e., illusions, passage hallucinations, and presence hallucinations with preserved insight) that over time can increase in frequency and severity and lead to loss of insight, disruptions in daily life, and the emergence of delusions); however, it is important to note that patients may experience minor phenomena that either resolve or remain relatively stable [6], [10]. PDP is associated with reduced health-related quality of life (HRQoL) and increased rates of extended hospitalizations, caregiver burden, morbidity, and long-term care placement [6], [11], [12].

Importantly, while PD is associated with increased mortality risk (MR) versus the general population [13], [14], the PDP MR is even higher than PD without psychosis [15], and this is independent of demographic and disease characteristics [4], [5], [6], [11], [12]. Furthermore, dementia risk is increased in patients with PDP, and the frequency of PDP is higher in PDD [16], [17]. A retrospective cohort study of Medicare claims (2008–2016) reported that the 2-year mortality rate in patients with dementia and psychosis was 52.0 % [18], which is substantially greater than the approximately 14 % to 25 % 2-year mortality rate in PDP [4].

Motor symptoms of PDP present an added challenge to the already progressive burden of PDP. Once PDP emerges, treatment of motor symptoms becomes challenging and quite different from the typical escalation of motor treatments over time seen in patients with PD without psychosis [6]. Often, motor therapies are not increased, despite increasing motor symptoms, and may be curtailed to reduce PDP symptoms, leading to worsening motor function [6]. Further, all currently available antipsychotics except pimavanserin have a higher affinity for D_2_ dopamine receptors, and current recommendations caution against the use of all antipsychotics (except clozapine, quetiapine, and pimavanserin) for PDP due to worsening of motor symptoms [19]. Only clozapine and pimavanserin have demonstrated efficacy for PDP in blinded, randomized trials [20]. However, clozapine requires regular blood monitoring for agranulocytosis and is therefore rarely used in the US [21], [22], [23]. In addition, off-target receptor antagonism by clozapine and quetiapine can worsen nonmotor symptoms of daytime somnolence and postural hypotension [21], [22]. Only pimavanserin has Food and Drug Administration (FDA) approval for the treatment of hallucinations and delusions associated with PDP [20], [24]. For these reasons, pimavanserin, which has a high selectivity for 5-HT_2A_ receptors and no significant affinity or functional activity at 5-HT_2B_, dopamine D2, or other monoaminergic receptors (except 5-HT_2C_), has been designated as an efficacious and clinically useful treatment for PDP that does not require specialized monitoring [25]. Prior to adding a preferred atypical antipsychotic for PDP, clinicians should first attempt a reduction in the amount and/or dosages of antiparkinsonian medications (i.e., anticholinergics and dopamine agonists) with the possible addition of an acetylcholinesterase inhibitor (i.e., rivastigmine); however, these reductions can lead to elevations in motor symptoms, and if psychotic symptoms remain despite these adjustments, the use of a preferred atypical antipsychotic should be considered [25].

The question of whether pimavanserin increases MR in PDP is very important. In 2005, on the basis of an increased MR in older adults with dementia-related psychosis treated with antipsychotics, the FDA issued a class boxed warning for atypical antipsychotic medications on the basis of studies conducted with olanzapine, aripiprazole, risperidone, and quetiapine [26]. The boxed warning was subsequently extended to typical antipsychotics in 2008 [27]. Later, a modified class boxed warning was included for pimavanserin on its approval in 2016 [28]. Since the warning was based on an increased MR associated with using off-label antipsychotics for symptoms of psychosis in older adults, who had diverse etiologies and dementia-related psychosis, compared with placebo, it is unknown whether there is an established risk for pimavanserin when prescribed for PDP with or without PDD [26], [27]. In Phase 3 studies, a higher MR was observed in patients with PDP treated with pimavanserin versus placebo, which resulted in a class boxed warning for pimavanserin [28], [29]. Pimavanserin is not FDA approved for patients with dementia who experience psychosis unless their hallucinations and delusions are related to PD [28].

### 1.1 Increased risk of mortality with PD

PD is associated with an increased MR versus the general population [13], [14] independent of PDP (Table 1) [29], [30], [31], [32], [33]. A meta-analysis of eight prospective observational studies reported the all-cause mortality rate of PD was more than twofold the general population (pooled analyses: risk ratio 2.22; 95 % CI, 1.78–2.77) [34]. Another meta-analysis, which evaluated 88 retrospective and prospective studies, reported PD mortality ratios of 0.9 to 3.8 versus a control population. In eight inception studies that recruited patients at or soon after diagnosis, the ratio for PD versus control patients was approximately 1.4-fold [35]. The increased MR in PD reflects several factors, including underlying neurodegenerative disease, aging, male preponderance, progressive motor symptoms, symptom severity (motor and nonmotor), and comorbidities [13], [14]. Despite recent advances in treatment, MR in patients with PD has reportedly either remained flat [13], [36] or increased [37].

**Table 1:** Postmarketing mortality rates of Parkinson disease and current treatment.

Data source	Mortality per 100 patient-years (95 % CI)
US Medicare data (2012–2015) [29], [33]	PD: 7.3 (7.15–7.47)
	PDP: 28.2 (27.5–28.8)
US Veterans Administration data [31]	Olanzapine: 29.3 (24.1–35.2)
	Quetiapine: 18.6 (16.9–20.3)
	Risperidone: 31.0 (26.4–36.1)
	Other atypical antipsychotics: 14.2 (7.6–24.3)
Acadia postmarketing data (April 29, 2016, to April 28, 2021) [30]	Pimavanserin: 15.4 (14.97–15.85)
Acadia placebo-controlled trials as of April 2018 [29], [32]^a^	Pimavanserin: 10.0
	Placebo: 10.9

Abbreviations: PD, Parkinson disease; PDP, Parkinson disease psychosis.

a These data include one study among patients with Alzheimer disease psychosis, which accounts for 90 of the 510 patients included in this rate calculation [29], [32].

### 1.2 Psychosis predicts increased mortality in patients with PD

**Fig. 1:**
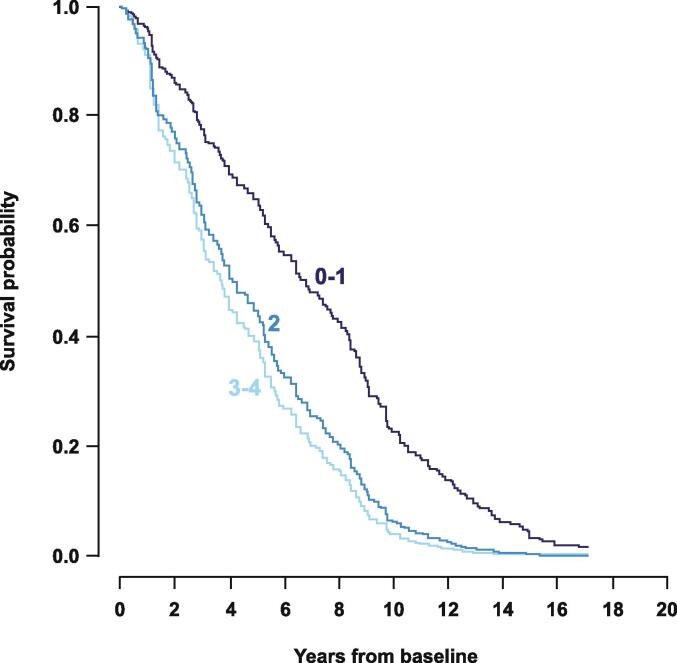
Predicted survival of patients with Parkinson disease according to baseline severity of psychotic symptoms by UPDRS I subscore, item 2 (thought disorder). UPDRS = Unified Parkinson’s Disease Rating Scale; 0–1 = no psychotic symptoms; 2 = hallucinations with retained insight; 3–4 = hallucinations or delusions without insight. Determined by Cox regression, adjusted to baseline age of 75 years. Reused with permission from Forsaa et al., 2010 [4].

Retrospective PD analyses have identified psychosis as an independent predictor of increased mortality [4], [35]. There is a substantial long-term MR in patients with PDP, and the mortality rate may be as high as 75 % at 7 years, even in less severe psychosis (i.e., Unified Parkinson’s Disease Rating Scale [UPDRS] I subscore of 2, indicating the presence of hallucinations with retained insight; Fig. 1) [4]. A large retrospective analysis of US patients (N > 50,000) [15] and a Medicare database (N = 106,893) [29], [30], [33] both reported that PDP was associated with an increased MR relative to patients with PD. Furthermore, a 2009 analysis of a US population–based cohort (N = 573) reported that the presence of hallucinations at PD diagnosis was a significant predictor of increased mortality [38].

The presence of dementia is a confounder of MR assessments in PDP and has been identified as a strong independent predictor of increased mortality [34], [35]. Although PDD is distinct from PDP, and is associated with an even higher MR, the conditions often occur together and are risk factors for each other, making it challenging to differentially assess the influence of dementia versus psychosis on PD’s MR [39], [40].

### 1.3 Pimavanserin clinical trials and postmarketing data related to mortality

Data from a phase 2/3 clinical trial demonstrated that pimavanserin improves symptoms of psychosis without worsening motor symptoms in patients with PDP; however, only short-term mortality rates are available because of the short study length (<6 weeks) [41], [42]. At the time pimavanserin was approved by the FDA (April 2016), one patient (0.4 %) had died while receiving placebo (of a cardiovascular event), whereas three patients (1.5 %) had died while receiving 34 mg pimavanserin (two patients died of infection and one of a cardiovascular event) [43]. All deaths were determined not to be due to the study drug per the investigators [44]. Furthermore, the crude mortality rate among patients treated with any dose of pimavanserin was 0.7 % (N = 420) [29], [30], [41], [42], [45].

In a postmarketing analysis of mortality events occurring between 2016 and 2021, the overall cumulative mortality rate for pimavanserin was 15.4 per 100 patient-years, with a minimum of 41,218 patients (30,426 patient-years) exposed (Table 1) [30]. The reported causes of death reflected common comorbidities in a population of PD patients treated for psychosis (i.e., PD disease progression, dementia, pneumonia, and respiratory and cardiac events) [30]. Studies suggest that atypical antipsychotic use is associated with increased mortality in PD, including a case-controlled study of patients aged 70 years and older, wherein typical and atypical antipsychotic use resulted in a higher risk of death in the 30 days after treatment initiation [46]. Another study reported that unadjusted mortality rates for atypical antipsychotics range from 14.2 to 31.0 per 100 person-years, which is comparable to or higher than the rates reported for pimavanserin (Table 1) [31]. In addition, mortality rates from placebo-controlled trials (as of April 2018) were 10.0 per 100 patient-years (1.2 %) among patients treated with pimavanserin (N = 510) and 10.9 per 100 patient-years (1.4 %) among patients treated with placebo (N = 357) [29], [32]. As such, the aim of this review was to summarize the real-world evidence about the MR and overall safety of pimavanserin relative to other atypical antipsychotics in patients with PDP.

## 2 Literature search methods

A PubMed search was conducted (from the beginning of records to July 27, 2023) using the following search terms: *pimavanserin* AND *antipsychotic* AND *mortality* AND *Parkinson’s disease* AND *psychosis*. We defined real-world evidence as data not collected during an initial double-blind clinical trial. As such we included retrospective analyses, observational studies, and/or open-label studies that were comparative studies of pimavanserin versus other atypical antipsychotics or a control group, comprised of patients not treated with antipsychotics or those not receiving pimavanserin. Studies that were reviews, expert commentaries, case reports, preclinical studies (i.e., pimavanserin’s mechanism of action), or studies of data from the FDA Adverse Event Reporting System were excluded.

## 3 Critical review of pimavanserin mortality studies

The literature search returned a total of 32 articles; of those, 22 articles not meeting the inclusion criteria were excluded. Of the remaining 10 retrospective, real-world, observational, and/or open-label study articles, 5 were comparisons of pimavanserin with other atypical antipsychotics, 3 were comparisons of pimavanserin with a control group of patients not treated with antipsychotics or not receiving pimavanserin, and 2 involved open-label extensions (OLEs) (Table 2).

**Table 2:** Studies of pimavanserin mortality in patients with Parkinson disease.

Study	Design	No. of patients	Primary objective	Mortality findings
*Real-world studies of pimavanserin* versus *other APs*				
Mosholder et al. 2022 [47]	Retrospective new-user cohort study of patients with PD in Medicare; April 2016 to March 2019	Total: 21,719	All-cause mortality with PIM vs AAPs	Mortality HR (95 % CI) for PIM vs AAPs:
		PIM: 3277		• Overall: 0.77 (0.66–0.90) • Treated 1–180 days: 0.65 (0.53–0.79) • Treated > 180 days: 1.05 (0.82–1.33)
		AAPs: 18,442		
Layton et al. 2022 [48]	Active comparator, new-user cohort study of patients with PD in Medicare; April 2016 to December 2019	Total: 21,975	All-cause mortality with PIM vs AAPs	Mortality HR (95 % CI) for PIM vs AAPs:
		PIM: 2892		• Overall: 0.78 (0.67–0.91)
		AAPs: 19,083		
Alipour-Harris et al. 2023 [49]	Retrospective study of new users with PD in Medicare; May 2016 to December 2018	Total: 3349	All-cause hospitalization and mortality with PIM vs quetiapine	Mortality-adjusted HR (95 % CI) PIM vs quetiapine:
		PIM: 844		• 90-day: 0.73 (0.48–1.13); *p* ≥ 0.05 • 180-day: 0.80 (0.58–1.10); *p* ≥ 0.05 • 1-year: 0.94 (0.74–1.19); *p* ≥ 0.05
		Quetiapine: 2505		
Horn et al. 2019 [50]	Single-center, retrospective cohort study in patients with PDP or DLB	Total: 92	Compare time to discontinuation in patients initiating PIM or quetiapine for psychosis	Mortality HR (95 % CI) for PIM vs quetiapine:
		PIM: 45		• 0.37 (0.06–2.45); *p =* 0.88
		Quetiapine: 47		
Nguyen et al. 2022 [51]	Retrospective new-user cohort study of patients with PD from commercial insurance database; May 2016 to March 2021	Total: 6635	All-cause mortality with PIM vs preferred or nonpreferred AAPs	Mortality-adjusted HR (95 % CI) for PIM vs preferred/nonpreferred AAPs:
		PIM: 775		• Preferred: 0.99 (0.81–1.20) • Nonpreferred: 0.98 (0.79–1.22)
		Preferred AAPs^a^: 4,563		
		Nonpreferred AAPs^a^: 1,297		

*Real-world studies of pimavanserin* versus *a control group*				
Longardner et al. 2023 [53]	Retrospective UCSD EHR study of PD; April 2016 to April 2019	Total: 315	Review of treatment regimen, clinical characteristics, and negative outcomes associated with increased mortality in patients with PDP	Mortality OR (95 % CI) vs untreated PDP controls:
		PIM: 34		• PIM: 0.171 (0.025–0.676); *p =* 0.026 • Quetiapine: 0.83 (0.405–1756); *p =* 0.624 • Both agents: 0.697 (0.277–1.716); *p =* 0.433
		Quetiapine: 147		
		Both agents: 68		
		Untreated: 66		
Moreno et al., 2018 [54]	Retrospective, new-user UCSD EHR study of PD; April 2016 to April 2018	Total: 676	Mortality with PIM, quetiapine, or both agents vs control	Mortality HR (95 % CI) for PIM, quetiapine, or both agents vs control:
		PIM: 113		• PIM: 1.23 (0.57–2.68); *p* ≥ 0.05 • Quetiapine: 1.74 (1.15–2.62); *p* < 0.05 • Both agents: 2.16 (0.93–5.01); *p* = 0.07
		Quetiapine: 505		
		Both agents: 58		
		Untreated: 784		
Hwang et al. 2021 [52]	Retrospective cohort study of patients with PD in long-term care using Medicare data; November 2015 to December 2018	Total: 20,398	Risk of hospitalization and death with PIM use	Mortality-adjusted HR (95 % CI) with PIM users vs PIM nonusers:
		PIM users: 2186		• 30-day: 0.76 (0.56–1.03); E-value,^b^ 1.96 • 90-day: 1.20 (1.02–1.41); E-value,^b^ 1.69 • 180-day: 1.28 (1.13–1.45); E-value,^b^ 1.88 • 1-year: 1.56 (1.42–1.72); E-value,^b^ 1.56
		PIM nonusers: 18,212		

*Open-label extension studies of PIM mortality*				
Ballard et al. 2020 [56]	Open-label extension study	PIM: 459	Assess the long-term safety and tolerability of PIM in patients with PDP	Observed MR: • PIM: 6.45 deaths per 100 patient-years
Ballard et al. 2015 [57]	Post hoc analysis of open-label extension study	Total: 423	Compare long-term safety in patients with PD taking PIM only vs those taking PIM and concomitant AAPs	MR:
		PIM only: 357		• PIM only: 4.5 deaths per 100 patient-years • PIM + AAPs: 18.8 deaths per 100 patient-years • PIM + AAP vs PIM only: IRR, 4.20; 95 % CI: 2.13–7.96
		PIM + AAPs: 66		

Abbreviations: AP, antipsychotic drug; AAP, atypical antipsychotic; DLB, dementia with Lewy bodies; EHR, electronic health record; HR, hazard ratio; IRR, incidence rate ratios; MR, mortality rate; OR, odds ratio; PD, Parkinson disease; PDP, Parkinson disease psychosis; PIM, pimavanserin.

a Preferred atypical antipsychotics included quetiapine and clozapine and non-preferred atypical antipsychotics included aripiprazole, asenapine, brexpiprazole, cariprazine, iloperidone, lumateperone, lurasidone, olanzapine, paliperidone, risperidone, or ziprasidone*.*

b E-values were calculated for sensitivity analyses to indicate the strength of association that an unmeasured confounder would need to have with both the exposure and the outcome variables, conditional on the measured covariates, to explain the observed association between the two variables entirely.

### 3.1 Retrospective, real-world studies of pimavanserin versus other atypical antipsychotics in PDP

Among the five studies comparing pimavanserin to other atypical antipsychotics, two large studies of a Medicare database of new antipsychotic medication users (i.e., “new users”) both reported comparable or lower all-cause mortality with pimavanserin versus other atypical antipsychotics (Table 2). The other three studies demonstrated comparable mortality between pimavanserin and other atypical antipsychotics.

A study by Mosholder et al., which enrolled new users (N = 21,719), reported that pimavanserin was associated with a lower all-cause MR compared with atypical antipsychotics both in the overall population (hazard ratio [HR] 0.77; 95 % CI, 0.66–0.90) and within 180 days of treatment (HR 0.65; 95 % CI, 0.53–0.79) [47]. The lower pimavanserin MR was not observed in nursing home patients, possibly because of the relatively small percentage of patients (approximately 15 % per group) and the high attrition beyond 180 days. In addition, among nursing home patients, the similarity in medical care among patients and the close attention to changes with treatment could partially explain the lack of a difference in MR between pimavanserin and atypical antipsychotics. Inverse probability of treatment weighting was used to balance treatment groups, accounting for chronic medical conditions, health care utilization, nursing home residence, medication classes, likelihood of mortality from comorbidities (by Charlson Comorbidity Index score), and frailty score. An analysis by Layton et al. also evaluated MR in new users with PDP based on 2016–2019 Medicare claims data (N = 21,975) [48]. Like Mosholder et al., they observed lower MR with pimavanserin versus atypical antipsychotics (HR 0.78; 95 % CI, 0.67–0.91) (Table 2) [48]. Unlike the Mosholder study, however, they did observe a lower MR for pimavanserin versus atypical antipsychotics (HR 0.78; 95 % CI, 0.60–1.01) in a long-term care/skilled nursing facility subgroup [47], [48]. The strengths of these two studies include the large sample size receiving pimavanserin and the nationally representative sample, which was restricted to probable PDP. Mosholder et al. also included methods to mitigate confounding differences between treatment groups. Both studies also contained limitations, which should be noted: for the comparisons with pimavanserin, both studies included patients receiving quetiapine (78 %) and those receiving non-preferred atypical antipsychotics, such as risperidone (9 %) or olanzapine (6 %). The study by Layton et al. included few patients receiving clozapine (<1%), and the study by Mosholder et al. did not included patients receiving clozapine as the authors state it is only available through a specialized program due to the risk for neutropenia. In addition, both studies were observational in nature, and confounding factors could still exist despite mitigation efforts. Although both studies are likely representative of the PDP population, differences in medication adherence (which was not measured) between treatment groups may have impacted the results.

Two relatively smaller studies that compared pimavanserin with quetiapine demonstrated similar MRs between the drugs [49], [50]. The study by Alipour-Harris et al. evaluated 3,394 new users with PD from a 15 % national sample of a Medicare claims database (2016 to 2018) and reported similar MRs between pimavanserin and quetiapine at 90 days, 180 days, and 1 year (Table 2) [49]. The model was adjusted for patient characteristics and stratified based on factors such as MR, frailty, and propensity scores. The other relatively small (N = 92) study by Horn et al. was a single-center cohort study of patients with PD or dementia with Lewy bodies (DLB) who initiated pimavanserin or quetiapine for psychosis. Consistent with the study by Alipour-Harris et al., this study also reported no significant difference in MR with pimavanserin versus quetiapine (HR 0.37; 95 % CI, 0.06–2.45; *p =* 0.88) [50]. Both studies have noteworthy limitations: they were relatively small (N = 3349 and N = 92), and although Alipour-Harris et al. adjusted for patient factors, other residual confounding factors may not have been captured. Furthermore, the study population may not have been broadly representative because it was based on a sample of only 15 % of the Medicare claims database [49]. In the study by Horn et al., some patients in the pimavanserin group were also taking quetiapine at some point during treatment, and the pimavanserin group had higher percentages of patients with DLB or who had previously received an antipsychotic drug [50].

A retrospective, new-user cohort study of patients with PD (N = 6635) from a commercial-insurance database reported a similar MR for pimavanserin versus preferred (i.e., quetiapine or clozapine) or nonpreferred (i.e., aripiprazole, asenapine, brexpiprazole, cariprazine, iloperidone, lumateperone, lurasidone, olanzapine, paliperidone, risperidone, or ziprasidone) atypical antipsychotics (mortality-adjusted HR 0.99; 95 % CI, 0.81–1.20) [51]. This study was strengthened by its relatively large sample size and the proportion of patients covered via Medicare Advantage; however, several limitations should be noted. The study population was from a claims database with predominantly commercially insured patients, who are often younger, whereas pimavanserin is more often prescribed to patients 65 years of age and older (i.e., Medicare beneficiaries). In addition, newer, more costly antipsychotic medications may be overrepresented in a commercially insured sample. Some of the mortality events may have been misattributed because the study allowed for switching of antipsychotic medications but assigned the mortality event attributed to the index drug [51]. Finally, it should be noted that studies of prescription claims do not necessarily accurately reflect medication use by patients [51].

### 3.2 Retrospective, real-world studies of pimavanserin versus a control group in patients with PDP

The literature search revealed four retrospective, real-world observational studies of the MR of pimavanserin versus either untreated control patients or control patients not receiving pimavanserin (Table 2). In terms of pimavanserin MR relative to a control group not receiving pimavanserin, one study reported a higher MR with pimavanserin [52].

Two studies utilizing the University of California San Diego (UCSD) Health Center’s electronic health record (EHR) data of pimavanserin versus an untreated control group reported MRs that were either lower [53] or similar [54] (Table 2). Longardner et al. (N = 315) reported that patients with PDP receiving pimavanserin had significantly lower MRs compared with untreated patients (odds ratio [OR] 0.171; 95 % CI, 0.025–0.676; *p* = 0.026) [53]. However, patients receiving quetiapine (OR 0.83; 95 % CI, 0.405–1.756; *p* = 0.624) or a combination of quetiapine and pimavanserin (OR 0.697; 95 % CI, 0.277–1.716; *p =* 0.433) had similar MRs to untreated patients [53]. There were no direct comparisons between quetiapine and pimavanserin. Moreno et al. (2018) measured mortality among 676 patients receiving pimavanserin (n = 113), quetiapine (n = 505), or both agents (n = 58) relative to age-matched, untreated control patients (n = 784) [54]. They reported an increased MR in the quetiapine group and a trend toward increased risk in the combination group (*p =* 0.07) but no increased MR in the pimavanserin group (HR 1.23; 95 % CI, 0.57–2.68; *p* ≥ 0.05). Limitations of these studies include their relatively small sample sizes (i.e., N = 315 and N = 676) and the comparison between treated and untreated patients, which likely represent different populations. In the Longardner et al. (2023) study, treated patients exhibited worse motor symptoms and more frequent nonmotor symptoms than untreated patients [53]. Both studies utilized EHRs from one institution (i.e., the UCSD Health System), which may have a specific patient population or prescribing practice that is different from the broader population of patients with PDP.

A retrospective study by Hwang et al. (2021), which included 20,398 Medicare patients with PD in long-term care, reported an increased MR with patients receiving pimavanserin (i.e. pimavanserin users) versus patients not receiving pimavanserin (i.e. pimavanserin nonusers) at 90 days (adjusted HR 1.20; 95 % CI, 1.02–1.41), which persisted after 180 days (adjusted HR 1.28; 95 % CI, 1.13–1.45) and up to 1 year (adjusted HR 1.56; 95 % CI, 1.42–1.72) [52]. However, there are several important limitations of that analysis. The comparison of pimavanserin users with presumed PDP to pimavanserin nonusers with an unknown PDP status introduces selection bias. Although the authors accounted for the presence of hallucinations and the extent of cognitive impairment, they did not have access to PD-specific disease severity data (i.e., Hoehn and Yahr or UPDRS), which restricted their ability to ensure consistency between the two groups [52]. Pimavanserin users also had more severe disease characteristics at baseline, and risks of 30-day hospitalization and 90-day mortality were not significantly different when groups were matched for baseline characteristics [52]. Finally, the study did not adjust for several confounding factors that are established contributors to increased MR in PDP (i.e., disease duration and age at onset of symptoms) [13], [52], [55].

### 3.3 Retrospective, open-label extension analyses of pimavanserin mortality

Two open-label analyses of pimavanserin mortality were identified; one was an open-label extension (OLE) study and the other a post hoc analysis of an OLE study (Table 2). The OLE study included patients who completed one of three previous placebo-controlled studies as well as one patient from a prior OLE study [41], [42], [56]. In this analysis over an 11-year period, 55.8 % of patients continued pimavanserin treatment for 1 year, and 18.1 % for 4 or more years [56]. Most deaths (76.3 %) occurred in patients aged 70 years and older, and the overall observed mortality rate was 6.45 deaths per 100 patient-years. An independent medical review of the 61 deaths did not find any to be drug-related; instead, they were consistent with the patients’ ages, advanced illness stage, and comorbidities [56].

The Ballard et al. (2015) study was a post hoc analysis of data from a multicenter, OLE study that evaluated long-term safety outcomes in patients with PDP taking pimavanserin with (n = 66) or without (n = 357) concomitant atypical antipsychotics [57]. There was a significant increase in MR in those taking concomitant atypical antipsychotics compared with pimavanserin alone (incidence rate ratio [IRR] 4.20; 95 % CI, 2.13–7.96). Patients taking concomitant atypical antipsychotics were also significantly more likely to experience serious adverse events (IRR 2.95; 95 % CI, 2.02–4.24). Indeed, after adjustment for time on follow-up, the MR in patients taking concomitant atypical antipsychotics was more than fourfold than that in patients taking pimavanserin alone. These results are consistent with previously reported findings regarding an increased MR and adverse events following treatment with atypical antipsychotics in patients with PDP [57]. This study was limited by the low number of patients taking concomitant atypical antipsychotics (n = 66) versus pimavanserin alone (n = 357), as well as the post hoc nature of the analysis.

## 4 Discussion

PDP is invariably progressive, and patients will require consideration of antipsychotic treatment once medical and medication-related triggers are evaluated and motor treatments are optimized [6], [58]. PDP treatment selection is important, and the need for psychosis symptom relief must be balanced with the need to minimize potential adverse events (i.e., motor function) [6], [58]. The atypical antipsychotic pimavanserin has a high selectivity for 5-HT_2A_ receptors and no significant affinity or functional activity at 5-HT_2B_, dopamine D2, or other monoaminergic receptors [59], which likely underlies its lack of impact on motor function.

The American Geriatrics Society’s (AGS) 2019 updated AGS Beers Criteria® for Potentially Inappropriate Medications in Older Adults states that generally, antipsychotics should be avoided for behavioral problems associated with dementia or delirium, except when behavioral interventions have failed or are not possible and when used for FDA-approved indications [19]. Among antipsychotics, only pimavanserin is approved by the FDA for the treatment of PDP hallucinations and delusions [22], [28], [61], [62]. In addition, the AGS recognizes only pimavanserin, clozapine, and quetiapine as acceptable antipsychotic medications in older adults with PD and states that pimavanserin and clozapine appear unlikely to result in worsening of PD [19]. This recommendation aligns with the International Parkinson and Movement Disorder Society Evidence-Based Medicine Committee’s recommendation that only pimavanserin and clozapine are efficacious and clinically useful and that clozapine requires specialized monitoring [60].

Our literature search identified 10 studies that met our inclusion criteria. Five studies included comparisons of pimavanserin with atypical antipsychotics [47], [48], [49], [50], [51], two were comparisons between pimavanserin and a control group of untreated patients [53], [54], one was a comparison of pimavanserin users to pimavanserin nonusers [52], and two were pimavanserin OLEs [57], [63]. Overall, the two Medicare database studies were likely the most rigorous and comprehensive, and both demonstrated comparable or lower all-cause MRs among new users of pimavanserin versus other atypical antipsychotics [47], [48]. Two other studies reported pimavanserin’s MR was similar to quetiapine [49], [50], and another reported the pimavanserin’s MR was similar to preferred/nonpreferred atypical antipsychotics [51].

While the study conducted by Hwang et al. reported an increased MR with pimavanserin, design limitations must be considered. The study compared pimavanserin users, who likely had PDP (which is known to increase MR), to pimavanserin nonusers with an unknown PDP status. Among pimavanserin nonusers only 22.1 % were taking other antipsychotics, potentially introducing selection bias [52]. Two UCSD EHR data studies demonstrated that the pimavanserin MR was either comparable to or significantly lower than that of untreated-control participants [53], [54]. The two OLE analyses by Ballard et al. reported pimavanserin mortality rates of 6.45 and 18.8 deaths per 100 patient-years [56], [57]. A limitation of these studies that should be considered is with respect to the unknown factors associated with patients who receive pimavanserin versus other guideline-recommended (i.e., quetiapine and clozapine) or non-recommended (i.e., risperidone and olanzapine) atypical antipsychotics. Overall, study designs and limitations should be considered when interpreting mortality findings from any of these analyses.

A prior systematic review and network meta-analysis by Yunusa et al. which evaluated the safety and tolerability of pimavanserin versus other atypical antipsychotics, reported that pimavanserin and clozapine significantly improved psychosis without worsening motor symptoms [64]. Indeed, that study and another systematic review both reported that pimavanserin demonstrated significant improvement versus placebo in psychosis symptoms as measured by scores on the Scale for Assessment of Positive Symptoms for Parkinson’s Disease Psychosis/Hallucinations and Delusions [64], [65]. In addition to improvements in nonmotor symptoms, a retrospective cohort analysis reported improvements in health care resource utilization for Medicare patients with PD treated with pimavanserin versus other atypical antipsychotics [66]. Those findings are also consistent with sensitivity analyses reporting a decreased risk of falls or fractures in patients receiving pimavanserin versus other antipsychotics, which primarily consisted of quetiapine or risperidone [67].

PD is associated with increased MR, which is further elevated in PDP [5], [6], [11], [12], [13], [14]. In addition, studies have reported patients with dementia and psychosis have increased rates of mortality (i.e. 2-year mortality rate of 52.0 %) [18], which has also been reported to be elevated in patients with PDP and dementia (i.e., 1-year mortality rate of approximately 15 %) [47]. All antipsychotics carry a boxed warning because of the risks of the drug class, but there are important differences among them [26], [27], and it is unclear what links, if any, exist between increased MR and atypical antipsychotic use in PDP. For pimavanserin, the reported MR appears similar to that for patients with PDP in general (Table 1). This analysis identified two large retrospective studies that both demonstrated comparable or lower all-cause mortality with pimavanserin versus other atypical antipsychotics [47], [48]. The findings from our literature review suggest that the MR associated with pimavanserin appears comparable to or lower than that for other atypical antipsychotics.

Pimavanserin’s risk–benefit profile is characterized by an improvement in psychosis without worsening motor symptoms [64] and lower rates of falls and fractures [67] with accompanying lower rates of hospital resource utilization [66]. When initiating any antipsychotic treatment in patients with PDP, physicians should assess a medication’s risk–benefit ratio as related to the expected impact on symptoms of psychosis, mortality, caregiver burden, and patient HRQoL and should monitor the risk of mortality, regardless of antipsychotic use. Further studies are warranted to examine long-term MRs specifically associated with antipsychotic treatment in this population.

## Funding information

This work was financially sponsored by Acadia Pharmaceuticals.

## CRediT authorship contribution statement

**Stuart H. Isaacson:** Writing – review & editing, Writing – original draft, Visualization, Validation, Supervision, Resources, Methodology, Investigation, Funding acquisition, Formal analysis, Data curation, Conceptualization. **Rajesh Pahwa:** Writing – review & editing, Writing – original draft, Visualization, Validation, Supervision, Resources, Methodology, Investigation, Funding acquisition, Formal analysis, Data curation, Conceptualization. **Fernando Pagan:** Writing – review & editing, Writing – original draft, Visualization, Validation, Supervision, Resources, Methodology, Investigation, Funding acquisition, Formal analysis, Data curation, Conceptualization. **Victor Abler:** Writing – review & editing, Writing – original draft, Supervision, Methodology, Data curation, Conceptualization. **Daniel Truong:** Writing – review & editing, Writing – original draft, Visualization, Validation, Supervision, Resources, Methodology, Investigation, Funding acquisition, Formal analysis, Data curation, Conceptualization.

## Declaration of competing interest

The authors declare the following financial interests/personal relationships, which may be considered as potential competing interests: Stuart Isaacson has received honoraria for CME and has served as consultant, received research grants, and/or acted as promotional speaker on behalf of AbbVie, Acadia, Acorda, Adamas, Addex, Allergan, Amarantus, Axovant, Biogen, Britannia, Eli Lilly, Enterin, GE Healthcare, Global Kinetics, Impax, Intec Pharma, Ipsen, Kyowa Kirin, Lundbeck, Michael J. Fox Foundation, Neurocrine, Neuroderm, the Parkinson Study Group, Pharma Two B, Roche, Sanofi, Sunovion, Teva, UCB, US WorldMeds, and Zambon. Rajesh Pahwa has received consulting fees from AbbVie, Acadia, Acorda, Adamas, Cynapsus, Global Kinetics, Lundbeck, Neurocrine, Pfizer, Sage, Sunovion, Teva Neuroscience, and US WorldMeds. He has received research grants from AbbVie, Adamas, Avid, Biotie, Boston Scientific, Civitas, Cynapsus, Kyowa Kirin, National Institutes of Health/National Institute of Neurological Disorders and Stroke, National Parkinson Foundation, and Parkinson Study Group. Fernando Pagan has been a speaker/consultant for Abbot Laboratories, AbbVie, Acadia, Acorda, Adamas, Amneal, Kyowa Kirin, Merz, Sunovion, Teva, and US WorldMeds. He has received research funding from the National Institutes of Health/National Institute on Aging, Sun Pharmaceutical Industries Ltd, and Alzheimer’s Research Foundation. He is a cofounder of and a shareholder in KeifeRx. Victor Abler is a salaried employee of Acadia Pharmaceuticals Inc. Daniel Truong has received research funding from AbbVie, Aeon, Biogen, Bukwang, Cerevel, Eli Lilly, Enterin, Ipsen, Kyowa Kirin, Lundbeck, Merz, National Institute of Neurological Disorders and Stroke, Neurocrine, Neuroderm, Parkinson’s Foundation, Revance, and Sunovion. He has received honoraria for consulting and speaker activities from Acorda, Amneal, Neurocrine, and US WorldMeds.
